# Chromosome-Level Assembly of the Chinese Hooksnout Carp (*Opsariichthys bidens*) Genome Using PacBio Sequencing and Hi-C Technology

**DOI:** 10.3389/fgene.2021.788547

**Published:** 2022-01-19

**Authors:** Xiaojun Xu, Wenzhi Guan, Baolong Niu, Dandan Guo, Qing-Ping Xie, Wei Zhan, Shaokui Yi, Bao Lou

**Affiliations:** ^1^ Institute of Hydrobiology, Zhejiang Academy of Agricultural Sciences, Hangzhou, China; ^2^ School of Life Sciences, Huzhou University, Huzhou, China

**Keywords:** *Opsariichthys bidens*, PacBio sequencing, Hi-C technology, chromosome-level assembly, *Opsariichthyinae*

## Introduction

Chinese hooksnout carp (*Opsariichthys bidens*) is an endemic Cypriniformes minnow in East Asia, and mainly distributed in China. Notably, this common minnow has undergone a long and complex taxonomic history. In 1960s, it was classified in Cyprinidae, Leuciscinae, *Opsariichthys* (Wu, 1964). With the advances on the application of molecular characters for the fish systematics in 1990s, *O. bidens* was assigned into Cyprinidae, Danioninae, *Opsariichthys* (Chen, 1998). Subsequently, its taxonomic status was revised several times (Mayden et al., 2009; Fang et al., 2009; Tang et al., 2010, 2013; Liao et al., 2011; Stout et al., 2016; Huang et al., 2017). According to the latest phylogenetic classification of bony fishes, *O. bidens* has been assigned into Xenocyprididae, Opsariichthyinae, *Opsariichthys* (Betancur-R et al., 2017), which has been adopted by the NCBI database (www.ncbi.nlm.nih.gov/Taxonomy) and FishBase (www.fishbase.org).

**FIGURE 1:**
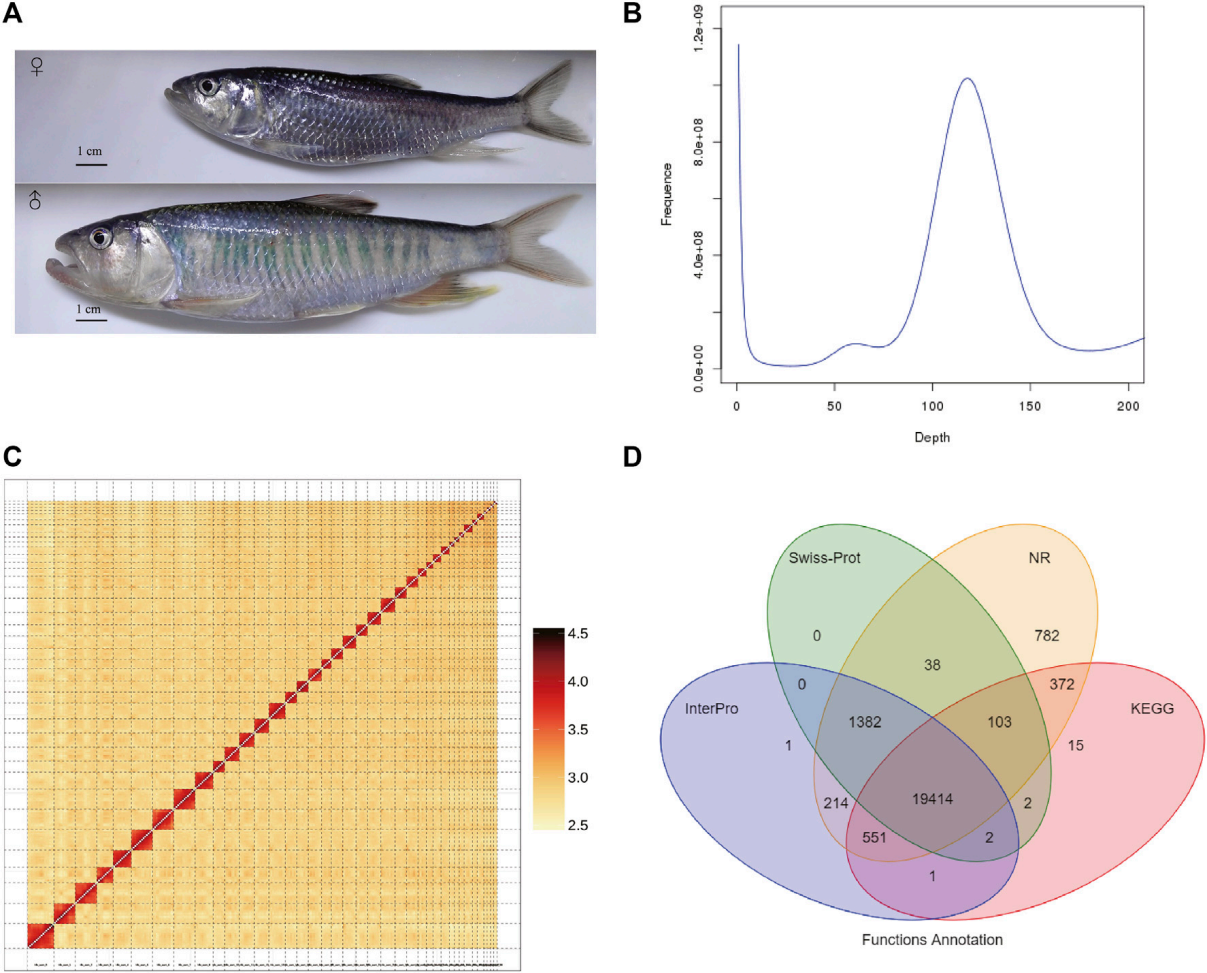
Genome assembly of *Opsariichthys bidens*. **(A)** The photo of female and male *O. bidens*; The body length and weight of female were 112 mm and 13.3 g, respectively; The body length and weight of male were 171 mm and 56.6 g, respectively. **(B)** The Kmer (K = 17) distribution of *O. bidens* genome. **(C)** The Hi-C heatmap used for integrating the scaffolds. **(D)** The Venn graph of the numbers of annotated genes with different databases.

For the desirable texture and flavor of the flesh, *O. bidens* has relatively high economic values. Artificial breeding of *O. bidens* began in 2008 (Jing, 2009), and the previous studies focused on the embryonic development (Jin et al., 2017), flesh nutrition content (Zhang Q. K. et al., 2019) and spermatogenesis (Tang et al., 2020) were reported in recent years. Due to the high price, disease resistance, and wide-range temperature adaptation, *O. bidens* (Figure 1A) has become an emerging commercial fish species.

Remarkably, *O. bidens* has obvious sex dimorphism (Lian et al., 2017). In aquaculture practice, the adult males are usually twice as large as the female siblings, and have gorgeous nuptial coloration, which brings to high ornamental property as a popular ornamental fish species. Hence, a high-quality genome sequence would facilitate the development of sex-specific markers and sex control breeding.

In this study, the chromosome-level assembly of *O. bidens* was constructed using PacBio sequencing and Hi-C technology. To the best of our knowledge, this is the sequenced genome with the largest chromosome number (2n = 78) in diploid Xenocyprididae (Arai, 2011). The genome resource will facilitate the studies of taxonomy, evolution, and genetic breeding of *O. bidens*.

### Data

A total of 135.07 Gb raw data were obtained from the Illumina X Ten platform for genome size estimation. The estimated genome size of *O. bidens* is about 899.69 Mb, and the heterozygous rate of genome was 0.36%. (Figure 1B). Meanwhile, 167.17 Gb long reads were generated by PacBio Sequel platform. The average length of long reads was 21,861 bp, and the N50 of long reads was 34,896 bp. The long reads were *de novo* assembled into 403 contigs with total length of 818.75 Mb. The N50 of the assembled contigs was 4.71 Mb and the largest contigs was 22.26 Mb in length.

We used BUSCO analysis to determine the completeness of genome assembly, and the result showed that this assembled genome contained 96.6% complete BUSCOs, including 91.1% complete and single-copy BUSCOs and 5.7% complete duplicated BUSCOs. Meanwhile, the evaluation using CEGMA showed that the completeness of assembly was 97.18%. After polishing with the Illumina short reads using NextPolish (Hu et al., 2020), the total length of assembled contigs was 818.75 Mb. The N50 of these contigs was 4.66 Mb.

Subsequently, 95.64 Gb Hi-C data was generated by Illumina NovaSeq 6000 platform and used for chromosome-level assembly. After quality control of Hi-C reads with HiCUP software (Wingett et al., 2015), a total of 2,210,719 valid pairs were detected and 95.66% unique Di-tags were obtained. With the Hi-C data, 82 contigs were anchored into 39 chromosomes with a total length of 814.71 Mb (Figure 1C), and the length of anchored chromosomes ranged from 6.77 to 42.84 Mb. Finally, the *O. bidens* genome was 818.78 Mb in length with N50 value of 25.29 Mb. To further validate the assembly completeness, we mapped the short reads to the final assembly, and the mapping rate was 98.76%.

A total of 42.39% of the genome (347.06 Mb) were identified as repetitive elements. The most abundant transposable elements (TEs) were long terminal repeats (LTRs, 35.12% of the genome), followed by DNA transposons (4.35%) and long interspersed elements (2.19%). Meanwhile, 23,992 protein-coding genes were annotated. The mean gene length was 16,469.11 bp. The average of CDS length was 1,670.54 bp, and the average number of exons per gene was 9.79. The comparative analysis of gene prediction with other fish species was performed (Figure S1). The function annotation of these protein-coding genes showed that 95.4% were annotated by at least one of the public databases (Figure 1D). Meanwhile, 1.07 Mb of the genome were annotated as ncRNAs, among which miRNA, tRNA and rRNA accounted for 0.084% of the genome. We performed the BUSCO analysis with the predicted protein-coding genes, and the result showed that a total of 93.5% complete BUSCOs were present with the gene annotation.

**FIGURE 2:**
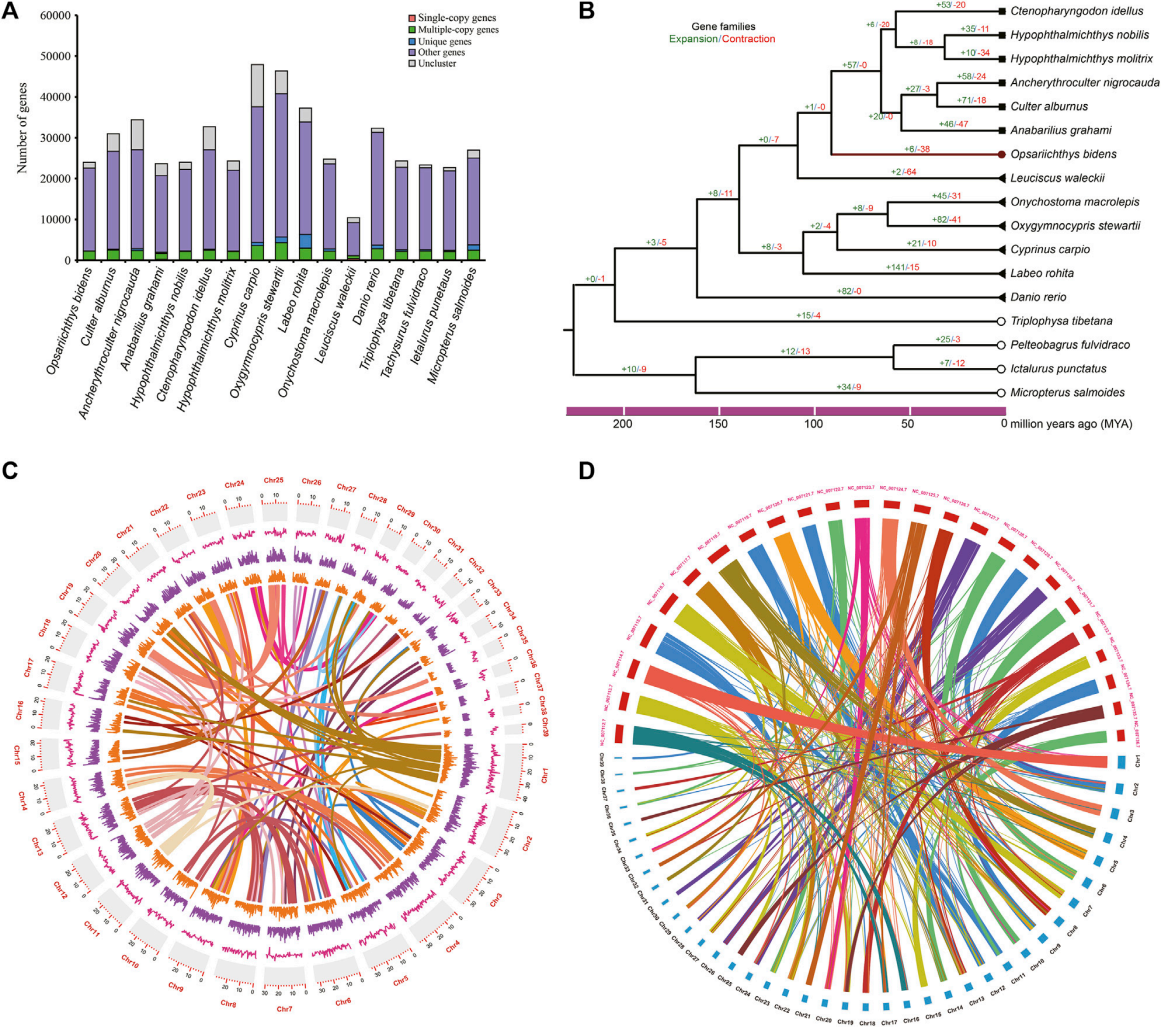
Comparative genome analyses. **(A)** the number of orthologous for 17 fish species and **(B)** the phylogenetic tree of 17 fish species; gene family expansions and contractions are indicated in green and red, respectively. **(C)** Synteny distribution of the 39 chromosomes of *O. bidens*; The tracks indicate the density of gene numbers and GC contents, respectively. **(D)** Comparative synteny analysis between *O. bidens* and Zebrafish.

The single-copy orthologous genes of 17 fish species were identified (Figure 2A), and the phylogenetic tree was constructed with 170 single-copy orthologous genes (Figure 2B), and the result showed that *O. bidens* was grouped with the species in families of Leuciscinae and Culterinae, indicating a closer relationship with these species. A total of 6 and 38 gene families significantly expanded and contracted in *O. bidens,* respectively. The expanded and contracted gene families contained 43 and 45 genes, respectively.

To further evaluate the quality of genome assembly, we compared *O. bidens* genome with zebrafish genome. The conservation synteny among the 39 chromosomes was shown in Figure 2C, and a total of 1,306 blocks were detected among the chromosomes. The gene synteny between *O. bidens* and zebrafish genomes is shown in Figure 2D. The chromosomes of *O. bidens* exhibited high homology with the zebrafish chromosomes, and several chromosomes of zebrafish were corresponding to two mini chromosomes of *O. bidens,* indicating that the large number of chromosome of *O. bidens* may originated from the chromosomal break of ancestral chromosomes.

## Materials and Methods

### Sampling, Library Construction, and Sequencing

A healthy female individual was collected from our fish base in Zhejiang Province, China. The muscle, blood, kidney, heart, brain, liver and ovary tissues were sampled and immediately frozen and stored in liquid nitrogen until extracting the genomic DNA and total RNA. High-quality DNA samples were extracted using the DNA Isolation Reagent Kit (TaKaRa, China) from muscle tissue. DNA quality and integrity was evaluated with 1% agarose gels. Firstly, a DNA sequencing library with insert size 350 bp was constructed following the instructions of Illumina DNA Prep kit. The library was sequenced on the Illumina HiSeq X Ten System using 150 bp paired-end mode in Novogene, Co. Ltd., Beijing. Meanwhile, PacBio SMRT libraries were prepared according to the manufacturer’s instructions, and the libraries were sequenced using a PacBio Sequel System. Additionally, total RNAs were extracted from the muscle, kidney, heart, brain, liver and ovary tissues using RNAiso kit (TaKaRa, China). The RNA sequencing library was constructed with the PacBio Iso-Seq Express Template Prep Kit 2.0 (Pacific Biosciences, United States) and sequenced using PacBio Sequel system. The Hi-C library was prepared from muscle tissue of the same individual following the standard protocol described previously (Belton et al., 2012). The constructed Hi-C library was sequenced with Illumina NovaSeq 6000 system.

### Genome Size Estimation, Genome Assembly and Polishing

The raw data generated by Illumina platform was filtered with fastp v0.20.0 program (Chen et al., 2018). Frequencies of *K*-mers (*K* = 17) were counted using Jellyfish (Marcais and Kingsford 2012). GenomeScope v1.0 (Vurture et al., 2017) was used to estimate size, repeat content and heterozygosity of the genome with maximum *K*-mer coverage of 10,000. The genome size was calculated as: size = *K*-mer number/peak depth. The genome assembly was performed using the FALCON assembler v2.1.0 (Chin et al., 2016), and the assembled contigs were polished with Illumina reads using NextPolish v1.4.0 software (Hu et al., 2020). The assembly completeness was evaluated by Core Eukaryotic Genes Mapping Approach (CEGMA) (Parra et al., 2007) and Benchmarking Universal Single-Copy Orthologs (BUSCO) v5.2.2 software (Simão et al., 2015) using the Actinopterygii geneset (v10.0). Subsequently, the Hi-C reads were aligned to the assembly using the Juicer v1.6.2 (Durand et al., 2016a). The contigs were ordered and anchored with Hi-C data using the allhic program (Zhang X. et al., 2019), and manually adjusted using the Juicebox Assembly Tools v1.11.08 (Durand et al., 2016b).

### Genome Annotation

Repetitive elements in the genome were identified using RepeatMasker (Chen, 2004) and RepeatModeler with default settings. The modeled repeats were classified into their subclasses using the Repbase v20.08 database (http://www.girinst.org/repbase/). Tandem Repeat was extracted using TRF (http://tandem.bu.edu/trf/trf.html) *ab initio* prediction. A custom library generated by a combination of Repbase and the *de novo* TE library which was processed by uclust to yield a non-redundant library was supplied to RepeatMasker for DNA-level repeat identification. Gene prediction was conducted through a combination of homology-based, *ab initio*, and transcript-based prediction methods. The full-length transcripts generated using PacBio Iso-Seq pipeline were used for transcript-based prediction. The transcripts were aligned to the genome using PASA program. Protein sequences of fish species including *Ctenopharyngodon idellus*, *Cyprinus carpio*, *Carassius auratus*, *Danio rerio*, and *Onychostoma macrolepis* were used as queries to search against the genome using tBLASTN. A *de novo* gene prediction was performed with Augustus v3.2.3 (Stanke et al., 2006), GlimmerHMM v3.04 (Majoros et al., 2004) and SNAP (Korf, 2004). The gene model was predicted by combination of three methods with EvidenceModeler v1.1.1 (Haas et al., 2008). Gene functional annotation was performed by aligning predicted protein-coding genes to the public databases using BLASTP and InterProScan70 v5.31 (Mulder and Apweiler, 2007), including NCBI NR, Swiss-prot, Pfam, Gene Ontology (GO), InterPro, and Kyoto Encyclopedia of Genes and Genomes (KEGG).

### Phylogenetic Analysis and Species Divergence Time Estimation

To investigate the phylogenetic status of *O. bidens*, we retrieved genome data of 16 fish species, including *Cyprinus carpio* (GenBank: GCA_000951,615.2), *Ictalurus punctatus* (GenBank: GCA_001660625.1), *Danio rerio* (GenBank: GCA_000002035.4), *Ancherythroculter nigrocauda* (NGDC: GWHAAZV00000000), *Micropterus salmoides* (GenBank: GCA_014851395.1), *Pelteobagrus fulvidraco* (GenBank: GCA_003724035.1), *Hypophthalmichthys molitrix* (CNGB: CNP0000974), *Hypophthalmichthys nobilis* (CNGB: CNP0000974), *Culter alburnus* (GenBank: GCA_009869775.1), *Oxygymnocypris stewartii* (GenBank: GCA_003573665.1), *Anabarilius grahami* (GenBank: GCA_003731715.1), *Labeo rohita* (GenBank: GCA_017311145.1), *Onychostoma macrolepis* (GenBank: GCA_012432095.1), *Leuciscus waleckii* (GenBank: GCA_900092035.1), *Triplophysa tibetana* (GenBank: GCA_008369825.1), and *Ctenopharyngodon idellus* (http://www.ncgr.ac.cn/grasscarp/) from public databases. All-to-all BLASTP was employed to identity the similarities among filtered protein sequences in these species with an E-value cutoff of 1e−5. We identified orthologous gene clusters using the OrthoMCL pipeline (Li et al., 2003). Protein sequences from the single-copy gene families were used for phylogenetic tree reconstruction. MUSCLE (Edgar, 2004) was used to generate multiple sequence alignments for protein sequences with default parameters, and the ambiguously aligned positions were trimmed using Gblocks (http://molevol.cmima.csic.es/castresana/Gblocks.html). The alignments of each family were concatenated to a super alignment matrix. The alignment matrix was used for phylogenetic tree reconstruction through maximum likelihood methods. The phylogenetic tree was constructed using RAxML v7.2.9 (Stamatakis, 2014) with 1,000 bootstrap replicates. Divergence time between species was estimated using MCMCtree with model of JC69 in PAML (Yang, 2007). The divergence time calibration of *Oxygymnocypris stewartii* and *Cyprinus carpio* were obtained from the TimeTree website (http://www.timetree.org/)*.* The likelihood analysis for gene gain and gene loss was identified using CAFE v4.2 (De Bie et al., 2006) with *p* < 0.05.

### Synteny Analysis

Synteny analysis of intra-genome was carried out using the MCScanX pipeline (Wang et al., 2012), output were converted to blocks by in-house Perl scripts. Circos (Krzywinski et al., 2009) was used to display the syntenic blocks. We identified syntenic blocks of genes between *O. bidens* and *D. rerio*. For the comparison, we carried out an all-to-all BLAST search of annotated protein sequences and ran MCScanX with the parameters “-s 10 -b 2”.

## Data Availability

The sequences of genome assembly are available in the National Genomics Data Center (NGDC) with accession number GWHBEIO00000000. The newick file of phylogenetic tree generated by RAxML is available in figShare with doi: https://figshare.com/articles/dataset/phylogenetic_tree_generated_by_RAxML/17085437/1. The karyotype image is available in figShare with doi: https://figshare.com/articles/figure/karyotype_image_of_O_bidens/17161865/1.

## Supplementary Material

Supplementary Figure S1 The comparative analyses of CDS length, exon length, exon number, gene length and intron length with 6 species. Cau, Cca, Cid, Dre, makouyu and Oma indicate Carassius auratus, Cyprinus carpio, Ctenopharyngodon idellus, Danio rerio, Opsariichthys bidens, and Onychostoma macrolepis, respectively.
